# Assessment and treatment of asylum seekers after a suicide attempt: a comparative study of people registered at mental health services in a Swedish location

**DOI:** 10.1186/s12888-015-0613-8

**Published:** 2015-10-07

**Authors:** Maria Sundvall, Dag H. Tidemalm, David E. Titelman, Bo Runeson, Sofie Bäärnhielm

**Affiliations:** Transcultural Centre, Stockholm County Council, Stockholm, Sweden; Department of Learning, Informatics, Management and Ethics, Karolinska Institutet, Stockholm, Sweden; Department of Clinical Neuroscience, Centre for Psychiatry Research, Karolinska Institutet, Stockholm, Sweden; National Center for Suicide Research and Prevention of Mental Ill-Health, Department of Learning, Informatics, Management and Ethics, Karolinska Institutet, Stockholm, Sweden; Department of Clinical Neuroscience, Karolinska Institutet, Stockholm, Sweden

**Keywords:** Refugees, Asylum seekers, Suicidal behaviour, Suicide risk assessment, Psychiatry, Health equity

## Abstract

**Background:**

Even though asylum seekers are considered vulnerable to mental ill-health, knowledge of their suicidal behaviour is limited. The aim of this study was to improve our understanding of factors that influence the clinical assessment of asylum seekers who have attempted suicide compared to the assessment of non-asylum seekers.

**Methods:**

The study focused on 88 asylum seekers registered for suicide attempts in mental health services 2005–2009, who were matched for age and gender and compared with 88 suicide attempters with Swedish personal identity numbers. The medical records were analysed with a quantitative protocol, focusing on social risk and protective factors, health history, current clinical picture as well as the assessment procedure, diagnostics, patterns of treatment and follow-up in this clinical group. Data was analysed using the chi-square test, Fisher’s exact probability test, and the Mann–Whitney *U* test.

**Results:**

As in earlier studies, asylum seekers were more traumatized, had different social risk factors and received different diagnoses than the controls. Asylum seekers were referred to less specialized follow-up after treatment, in spite of their health history and of previous and current clinical pictures indicating a similar or - in the case of the female asylum seekers - more serious mental health condition. Female asylum seekers also received more intense and prolonged in-patient treatment than female controls.

Asylum seekers appeared to have social networks more often than the control group. However, there was less documentation of the social context, previous suicidal behaviour, and on suicide in the family and close environment of the asylum-seeking men. Information on suicidal intent was lacking in a majority of both groups.

The time relation of the suicide attempt and the asylum process suggested the importance of the asylum decision, as well as the possible role of earlier mental health problems and premigration stress, for the suicidal behaviour.

**Conclusions:**

The groups had different sets of risk factors and clinical pictures. There was a lack of early and thorough exploration of suicide intent for both groups, and of contextual and subjective factors for the asylum seekers. Differences in follow-up indicate unequal access to care.

## Background

Asylum seekers are individuals who have applied for protection as refugees in a foreign country but who have not yet received an answer to their application for asylum. They are considered vulnerable to mental ill-health, since they are exposed to multiple and often recent stressors before as well as after having migrated, and since their legal status in the host country is uncertain [1]. Assessment of suicide risk and diagnostics in asylum seekers may be particularly complicated due to language barriers as well as to migration-related, cultural and legal factors. There is limited knowledge of how the asylum seeker’s specific contextual situation affects his or her mental health, including suicidality, and the encounter with psychiatric care.

Recent European studies of asylum seekers show increased rates of suicide, especially among male asylum seekers, and increased suicidal behaviour compared with the general population, with differences in risk related to region of origin, gender, and age [2–6]. Asylum seekers have high levels of psychological distress, such as PTSD, depression and anxiety [7, 8]. In some studies asylum seekers have even higher risks than the broader group of refugees [8, 9], who in turn also have poorer mental health and increased risk of suicidal behaviour [1, 10–16]. An exception is a Swedish study of refugees with PTSD in which there was no difference in suicidal behaviour between the asylum seekers and those who had received a permanent residence permit [17].

The increased risk has mainly been explained by postmigration factors, which differ between asylum seekers and other groups of immigrants [18]. Postmigration factors that affect the risk of suicidal behaviour in asylum seekers include:

- The asylum process, especially the rejection of an asylum application, a long waiting time, receiving temporary permits, financial problems and not being able to work, being kept in detention [2–4, 7, 18–22];
- Trauma (including torture), especially trauma leading to the development of PTSD [23, 24];
- Relationship issues and loss of family members [2, 3, 22, 25, 26].

Asylum seekers often have difficulties accessing health care. In many countries their right of access is legally limited and they may also abstain from seeking help for fear of being deported [27, 28]. In Sweden access to care for asylum seekers is legally restricted to “treatment that cannot wait”, a rule that has been interpreted in different ways in different clinical contexts and time periods.

When asylum seekers seek care, there are additional obstacles to the encounter including language barriers [29]. Assessing suicidal intention is an essential, yet delicate task that requires a trustful dialogue, which may be difficult to establish with asylum seekers. Guidelines in Sweden recommend specific scales as a complement to the clinical interview [30], but there is conflicting evidence on their validity [31–33].

The psychiatric consultation is also influenced by legal aspects [34]. Psychiatrists are often requested to write certificates regarding the mental health status of the asylum seeker after a suicide attempt, certificates to be used in the ongoing legal asylum application process.

Asylum seekers have been shown to receive lower-grade treatment than that received by comparison groups in the population [2, 4, 35]. In the period 2005 to 2009 asylum seekers in Sweden had more registered care episodes and more days in inpatient psychiatric treatment than the general population [36–40].

Systematic knowledge on suicidal behaviour of asylum seekers is still limited. In Sweden asylum seekers are difficult to follow up in research and health care, since they are not included in national population or health registers, and since asylum seekers often live in private accommodation, and not in asylum centres.

The overarching aim of this study was to increase knowledge of the clinicians’ assessments of suicide risk in asylum seekers, both related to what they build their assessments on and what they lead to. Our specific aims were to identify differences in this respect between asylum seekers and patients with citizenship or permanent residence, all of whom had attempted suicide. The following variables were investigated:

- Patient characteristics that inform suicide risk assessment, including social risk and protective factors, health history and history of suicidal behaviour, clinical picture at assessment, gender differences.
- Aspects of the encounter with the mental health services including the assessment procedure, diagnostics, treatment and follow-up.

## Methods

### Subjects

The material consisted of the medical records kept by clinicians when patients were registered for a suicide attempt from 2005 to 2009 and treated at the central emergency services of the Stockholm County mental health services. The catchment area of this unit includes about two million inhabitants.

From the registrations, patients without Swedish personal id numbers were selected and reading the medical records made it possible to identify 93 asylum-seeking patients among them. Out of these 93 patients 88 were found to be eligible for the study. Five individuals had been misclassified as suicide attempters and were excluded from the sample.

From the same registrations we created a control group of 88 patients, who were not asylum seekers, with a Swedish personal identity number, Swedish- or foreign-born, with or without Swedish citizenship. The controls were matched in gender, five-year age class and year of registration for suicide attempt.

### Setting

At the time of the registrations, the Central emergency service was the only emergency unit in the Stockholm County, and most persons with suicidal behaviour during out-of-office hours without ongoing contact in the mental health services were assessed there.

### Procedure and instruments

The medical records were analysed with a standardized protocol, based on a literature review of earlier research on risk factors for suicide and mental ill health in general, including earlier somatic and mental health and substance abuse, previous suicide attempts, suicide in the family, trauma, social isolation [41], social network as a protective factor, as well as risk factors relevant for asylum seekers including factors related to the asylum procedure. We added variables describing the encounter with health care. Traumas and other stressful situations were divided into having occurred before and after arriving in Sweden and into different types of traumas and other stressful situations (see Table 2). Symptoms were noted irrespective of whether they were documented in the medical history, the history of the present illness or in the examination of the patient and irrespective of whether the clinician had named them or not. Diagnoses were registered in three forms: according to DSM-IV, according to ICD-10 or as tentative descriptive assessments.

In order to measure suicide intent we applied the 15-items version of the Suicide Intent Scale, SIS, to the medical records. SIS is divided into one section with objective questions about circumstances of the suicide attempt and one with questions about the individual’s subjective intent. Suicide methods were categorized as either self-injury or self-poisoning [42].

### Characteristics of data

The information we were able to find in the medical records varied from ample information on current health problems and the current care episode to scarce documentation about some of the variables, especially ethnicity, country of birth of mother and father, number of siblings, traumas suffered by family members, earlier occupation before arriving in Sweden, suicide or deaths in family. Due to the missing data most of these variables were excluded from the analysis.

Data on the variables derived from the Suicide Intent Scale were missing in the vast majority of cases for both asylum seekers and controls. There were only five of the fifteen variables about which information was found in about 50 % or more of the sample: carrying out the suicide attempt with someone else present (information available in 59.7 % of the subjects), purpose of suicide attempt being death (48.3 %), considering the act a serious attempt to end life (60.2 %), attitude towards living/dying (51.7 %) and degree of premeditation (54.5 %). We restricted the comparative analysis to these five items.

Due to the nature of medical records, and how they are kept by clinicians, it was most often not possible to distinguish an actual absence of a sought-for phenomenon from data missing because of non-documentation. They were then registered as missing.

### Data analysis

Comparisons between the asylum seekers and the control group were made for those variables in the protocol where there was a sufficient level of information found in the medical records.

Variables were grouped together to constitute meaningful categories, for instance past traumas and earlier mental health problems. Bivariate analyses were conducted to make the comparisons. In these analyses, differences in proportions were tested in two-by-two tables with the chi-square test and Fisher’s exact test; the Mann–Whitney *U* Test was used to test differences in continuous variables. Apart from frequencies and proportions, descriptive data included means, ranges and medians for those variables where this was appropriate. Data were analysed for the whole group as well as separately for each gender, comparing male and female asylum seekers respectively with their gender in the control group. SPSS for Windows v. 22.0 (SPSS Inc.) was used for the analyses.

Ethical approval was given by the Regional Ethical Review Board of Stockholm (number 2010/3:6 and 2012/982-32).

## Results

### Characteristics of study population

The asylum seekers were 47 men and 41 women between 18 and 54 years of age with a median age of 29. The mean age of the asylum seekers was 31.3 (SD 8.7). The mean age of the asylum - seeking men was 32.3 (SD 9.5) and of the asylum - seeking women 30.0 (SD 7.6).

Their most common countries of birth were Bangladesh (*n* = 20), Iraq (*n* = 14), Iran (*n* = 12) and Azerbaijan (*n* = 5). For the women the most common country of birth was Bangladesh, for the men Iraq. See Table 1.

**Table 1:** Countries of birth of asylum seekers

Number of asylum seekers	Birth country
20	Bangladesh
14	Iraq
12	Iran
5	Azerbaijan
4	Russia
3	Afghanistan, Egypt, Syria, Uzbekistan
2	Ethiopia, Jordan, Congo^a^, Pakistan
1	Armenia, Eritrea, Georgia, Yugoslavia, Kazakhstan, Kosovo, Lebanon, Libya, Mongolia, Senegal, Sierra Leone, Somalia, Turkey

Names of countries are given as in the medical records

^a^Probably DR Congo

In the control group, which was matched for age and gender, 22 persons were documented as foreign-born. Four of them were documented as adopted in childhood by families in Sweden. It is not possible to know from the medical records whether or not all of the other 66 were born in Sweden.

Of the asylum seekers, 9.1 % were documented as having lived in Sweden for half a year or less, 52.1 % for two years or less and 6.8 % for longer than five years.

### Patient characteristics informing suicide risk assessment

Of the asylum seekers, 39.1 % made the registered suicide attempt after a recent rejection of the claim for political asylum, but 24.1 % made the attempt in the first period of asylum seeking, while still waiting for the official decision. More women among the asylum seekers made their suicide attempt after a recent rejection.

There were similarities between the asylum seekers and the control group with regard to their previous health histories of earlier somatic or mental health problems, previous suicide attempts and the proportion of individuals assessed as suicidal after previous self-destructive behaviour. Even before arriving in Sweden mental health problems were reported in 44.3 % of the asylum seekers, and a majority of them (28.4 %) had sought care for these problems.

The asylum seekers were significantly more often exposed to trauma and significantly more often had PTSD-related (44.3 vs. 5.7 % for the controls, *p* <0.001) or psychosis-like symptoms (27.6 vs. 11.4 % for the controls, *p* <0.01) and symptoms related to psychosocial stress, such as sleep disturbances, pain, and appetite or weight problems (mean rank 102.2 vs. 74.9 for the controls, *p* <0.001). Female asylum seekers had a slightly higher documented level of trauma than the male asylum seekers (see Table 2). The Bangladeshi asylum seekers were significantly more often exposed to torture than asylum seekers with other countries of birth (66.7 vs. 13.6 %, *p* <0.001).

**Table 2:** Trauma and stressful situations in asylum seekers and control group (*n*, %)

Category	Men, asylum seekers	Men, control group	Women, asylum seekers	Women, control group	Total, asylum seekers	Total, control group
Trauma						
Persecution, harassment	32 (68.1)*	1 (2.1)	23 (56.1)*	2 (4.9)	55 (62.5)*	3 (3.4)
Threats	14 (29.8)*	3 (6.4)	12 (29.3)*	2 (4.9)	26 (29.5)*	5 (5.7)
Prison/lawful punishment, political reasons	4 (10.3)	0 (0.0)	5 (13.9)	0 (0.0)	9 (12.0)	0 (0.0)
Prison/lawful punishment, all reasons	12 (25.5)*	4 (8.5)	8 (19.5)	3 (7.3)	20 (22.7)*	7 (8.0)
War events as military staff/as civilians	9 (19.1)*	2 (4.3)	3 (7.3)	0 (0.0)	12 (13.6)*	2 (2.3)
Assault, robbery	8 (17.0)	7 (14.9)	13 (31.7)	12 (29.3)	21 (23.9)	19 (21.6)
Torture	14 (35.0)	0 (0.0)	6 (16.2)	0 (0.0)	20 (26.0)	0 (0.0)
Abuse before age of 18^a^	0 (0.0)	4 (8.5)	7 (17.1)	10 (24.4)	7 (8.0)	14 (15.9)
Rape, abuse, kidnapping^a^	2 (4.3)	2 (4.3)	17 (41.5)*	7 (17.1)	19 (21.6)*	9 (10.2)
Non-specified trauma	2 (4.3)	1 (2.1)	6 (14.6)	1 (2.4)	8 (9.1)	2 (2.3)
Total exposure to trauma	39 (83.0)*	13 (27.7)	36 (87.8)*	19 (46.3)	75 (85.2)*	32 (36.4)
Stressful situations						
Alcohol/substance abuse or disease in parents	0 (0.0)	12 (25.5)*	1 (2.4)	16 (39.0)*	1 (1.1)	28 (31.8)*
Socioeconomic conditions in childhood and youth	3 (6.4)	6 (12.8)	3 (7.3)	6 (14.6)	6 (6.8)	12 (13.6)
Bullying	1 (2.1)	4 (8.5)	0 (0.0)	7 (17.1)*	1 (1.1)	11 (12.5)*
Family conflict	1 (2.1)	5 (10.6)	11 (26.8)	5 (12.2)	12 (13.6)	10 (11.4)
Relational conflict	3 (6.4)	25 (53.2)*	4 (9.8)	16 (39.0)*	7 (8.0)	41 (46.6)*
Recent relational conflict	1 (2.1)	14 (29.8)*	0 (0.0)	4 (9.8)	1 (1.1)	18 (20.5)*
Loss	7 (14.9)	7 (14.9)	10 (24.4)	8 (19.5)	17 (19.3)	15 (17.0)
Migration related, other than asylum process	1 (2.1)	2 (4.3)	3 (7.3)	1 (2.4)	4 (4.5)	3 (3.4)
Socio-economic stress	11 (23.4)	15 (31.9)	11 (26.8)	16 (39.0)	22 (25.0)	31 (35.2)
Work/study related	0 (0.0)	11 (23.4)*	2 (4.9)	8 (19.5)*	2 (2.3)	19 (21.6)*
Health related	4 (8.5)	4 (8.5)	7 (17.1)	4 (9.8)	11 (12.5)	8 (9.1)
Circumstances concerning own children	3 (6.4)	2 (4.3)	12 (29.3)	7 (17.1)	15 (17.0)	9 (10.2)
Other	0 (0.0)	1 (2.1)	2 (4.9)	9 (22.0)*	2 (2.3)	10 (11.4)*
Phase in the asylum process						
Waiting for decision	12 (25.5)	-	9 (22.0)	-	21 (23.9)	-
Recent rejection	17 (36.2)	-	17 (41.5)	-	34 (38.6)	-
Have appealed decision	9 (19.1)	-	8 (19.5)	-	17 (19.3)	-
Earlier asylum seeker, still staying in the country after rejection and appeal	1 (2.1)	-	3 (7.3)	-	4 (4.5)	-
Other or unclear situation	8 (17.0)	-	4 (9.8)	-	12 (13.6)	-

^a^Abuse defined as physical abuse, sexual abuse, emotional abuse and neglect

* = significant difference, *p*-value < 0.05

The asylum-seeking women were significantly more often married or had a partner and more often had children than the women in the control group. For more information on social networks, see Table 3.

**Table 3:** Social networks in asylum seekers and control group (*n*, valid %)

Social networks	Men, asylum seekers	Men, control group	Women, asylum seekers	Women, control group	Total, asylum seekers	Total, control group
Married/in cohabitation/partner	19 (50.0)	15 (32.6)	25 (61.0)*	15 (36.6)	44 (55.7)*	30 (34.5)
Children	18 (51.4)	16 (34.8)	26 (65.0)*	17 (41.5)	44 (58.7)*	33 (37.9)
Primary family in another country	19 (86.4)*	3 (17.6)	27 (100.0)*	3 (16.7)	46 (93.9)*	6 (17.1)
Secondary family in another country	11 (33.3)*	1 (2.7)	13 (32.5)*	2 (6.7)	24 (32.9)*	3 (4.5)
Private accommodation	27 (57.4)	41 (87.2)	34 (82.9)	37 (90.2)	61 (83.6)	78 (88.6)
Living in a single household	7 (20.6)	20 (43.5)*	2 (5.0)	20 (50.0)*	9 (12.2)	40 (46.5)*
Living with family/relatives	13 (38.2)	24 (52.2)	25 (62.5)*	17 (42.5)	38 (51.4)	41 (47.7)
Current social contacts	28 (59.6)	29 (61.7)	29 (70.7)	22 (53.7)	57 (64.8)	51 (58.0)
Currently studying/working	7 (14.9)	28 (59.6)*	9 (22.0)	20 (48.8)*	16 (18.2)	48 (54.5)*

* = significant difference, *p*-value <0.05

The asylum-seeking men had significantly more often been treated in primary care for previous mental health problems than the male controls (68.0 vs. 12.5 %, *p* <0.001).

The controls were significantly more often living alone, had more documented stressful situations other than trauma, significantly more often documented alcohol and substance abuse in their medical history than the asylum seekers (39.5 vs. 5.8 % for alcohol abuse, *p* <0.001; 35.2 vs. 2.3 % for drug abuse, *p* <0.001) as well as in their clinical picture at assessment. Thirty-three of the controls (37.5 %) were actually documented as under the effect of alcohol or drugs at the assessment, whereas the same was true for only one asylum seeker (a man).

The women in the control group had significantly more often documented earlier violent behaviour than the asylum-seeking women (26.8 vs. 7.3 %, *p* <0.05). They were also significantly more often treated in out-patient mental health services for previous mental health problems, including previous suicidal behaviour, compared to the female asylum seekers (95.1 vs. 71.9 %; *p* <0.01).

### The encounter with mental health services at the registered care episode

The use of an interpreter for the first suicide risk assessment interview was only noted in 35 of the 88 cases. However, this may be due to non-documentation and the real figure is probably higher. The use of staff or relatives as interpreters was noted in only a few cases.

Assessments at the registered care episode differed between the two groups. For the asylum-seeking men there was significantly less documentation of their social context including civil status, household composition, earlier and current occupation, and current social contacts. There was also significantly less information compared to the men in the control group on previous suicide attempts (missing information in 12.8 vs. 0.0 %, *p* <0.05) and on suicide in the family and close environment (missing information in 78.7 vs. 40.4 %, *p* <0.001).

In the assessment of suicidal behaviour, there was neither significant difference in the number of asylum seekers and controls who were assessed by a specialist in psychiatry within 24 h, nor in the number of cases in which a specialist in psychiatry was consulted within 24 h.

Of the recorded assessments of suicidal intent, a significantly higher proportion of the asylum seekers reported death as the purpose of the attempt (93.0 vs. 73.8 %, *p* <0.05) and considered the act as a serious attempt to end life (97.8 vs. 65.0 %, *p* <0.001). A significantly higher proportion of the controls (25.5 vs. 53.7 %, *p* <0.01) had carried out their suicide attempts without anyone else present. Significantly more female asylum seekers than female controls had used a suicide method characterized as self-injury. There was a difference related to country of birth, with Bangladeshi patients using hanging, strangling and suffocation significantly more often than asylum seekers with other countries of birth (50.0 vs. 20.6 %, *p* <0.01). When we separated for gender among the Bangladeshi patients, the differences were no more significant, indicating a lack of statistical power. However, even when we excluded the Bangladeshi patients, the asylum seekers significantly more often used hanging, strangling or suffocation than the controls (20.6 vs. 8.0 %, *p* <0.05). The difference was significant for both men and women. For different suicide methods, see Tables 4 and 5.

**Table 4:** Methods of intentional self-harm (ICD-10 codes) in asylum seekers and control group (*n*)

Methods of self-harm	Asylum seekers	Control group
X60 Self-poisoning, nonopiod analgesics, antipyretics, antirheumatics	3	0
X61 Self-poisoning, antiepileptic, sedative-hypnotic, antiparkinsonism, psychotropic drugs not elsewhere classified	16	7
X64 Self-poisoning, other and unspecified drugs, medicaments, biological substances	13	35
X65 Self-poisoning, alcohol	0	1
X67 Self-poisoning, gases and vapours	0	2
X69 Self-poisoning, other and unspecified chemicals, noxious substances	1	0
X70 Hanging, strangulation, suffocation	24	7
X71 Drowning, submersion	2	1
X72 Handgun discharge	1	1
X73 Rifle, shotgun and larger firearm discharge	0	1
X78 Sharp object	14	29
X80 Jumping from a high place	19	12
X81 Jumping or lying before moving object	8	0
X82 Crashing of motor vehicle	0	2
X83 Other specified means	2	1
X84 Unspecified means	1	0
Total	104	99

The table lists the number of suicide methods used at the recent suicide attempt, which explains why there are more methods than subjects (individuals may have used more than one method)

**Table 5:** Suicide method categorized as self-injury or self-poisoning

Suicide method	Men, asylum seekers	Men, control group	Women, asylum seekers	Women, control group	Total, asylum seekers	Total, control group
Self-injury	35 (74.5)	34 (72.3)	26 (63.4)*	17 (41.5)	61 (69.3)	51 (58.0)
Self-poisoning	12 (25.5)	13 (27.7)	15 (36.6)	24 (58.5)	27 (30.7)	37 (42.0)

Number of subjects using any method categorized self-injury (X67, X70–84) compared with subjects using only methods categorized as self-poisoning (X61–66, X68–69) (*n*, %)

* = significant difference, *p*-value <0.05

Summing up the different diagnostic groups in ICD-10 and DSM-IV, mood disorder was the most common diagnosis for both groups. Asylum seekers were significantly more often diagnosed as having PTSD. Female asylum seekers significantly more often received the diagnoses stress reactions and adjustment disorders than female controls. The control group was more often diagnosed as having personality disorder and alcohol and substance abuse. See Table 6.

**Table 6:** Diagnosis given at registered care episode (*n*, %)

Diagnostic group	Men, asylum seekers	Men, control group	Women, asylum seekers	Women, control group	Total, asylum seekers	Total, control group
Psychotic disorder	1 (2.1)	3 (6.4)	1 (2.4)	0 (0.0)	2 (2.3)	3 (3.4)
Alcohol and substance abuse	0 (0.0)	3 (6.4)	0 (0.0)	4 (9.8)	0 (0.0)	7 (8.0)*^a^
Mood disorder	12 (25.5)	13 (27.7)	8 (19.5)	7 (17.1)	20 (22.7)	20 (22.7)
Anxiety disorder	2 (4.3)	2 (4.3)	0 (0.0)	1 (2.4)	2 (2.3)	3 (3.4)
Stress reactions	9 (19.1)	6 (12.8)	10 (24.4)*	3 (7.3)	19 (21.6)	9 (10.2)
Adjustment disorder	4 (8.5)	2 (4.3)	9 (22.0)*	1 (2.4)	13 (14.8)	3 (3.4)
PTSD	6 (12.8)	1 (2.1)	6 (14.6)*^a^	0 (0.0)	12 (13.6)	1 (1.1)
Dissociation	0 (0.0)	0 (0.0)	1 (2.4)	0 (0.0)	1 (1.1)	0 (0.0)
Personality disorder	1 (2.1)	4 (8.5)	0 (0.0)	6 (14.6)*^a^	1 (1.1)	10 (11.4)*
Eating disorder	0 (0.0)	1 (2.1)	0 (0.0)	1 (2.4)	0 (0.0)	2 (2.3)
Developmental disorder	0 (0.0)	0 (0.0)	0 (0.0)	2 (4.9)	0 (0.0)	2 (2.3)
Other	0 (0.0)	1 (2.1)	1 (2.4)	2 (4.9)	1 (1.1)	3 (3.4)
No diagnosis given	8 (17.0)	10 (21.3)	5 (12.2)	6 (14.6)	13 (14.8)	16 (18.2)

DSM-IV and ICD-10 diagnosis are combined together and divided into diagnostic groups. One individual can have more than one diagnosis. The tentative written diagnoses were not included in the diagnostic groups in the table; this explains why the sums of the columns are less than 100 %

PTSD is registered separately from anxiety disorders (to which it belonged in the DSM-IV)

* = significant difference with the Pearson Chi-square test or Fisher’s exact test, *p*-value <0.05

^a^Cells had expected count less than 5; therefore, Fisher’s exact test was used instead of the chi-square test

Among the participants with documented trauma, more asylum seekers than controls received the diagnosis PTSD, but the difference was not significant. Among the 20 asylum seekers who were exposed to torture, 7 received a diagnosis of PTSD.

Significantly more asylum seekers than controls were hospitalized either at the local mental health services or sent to other units in the county for hospitalization. The association was significant for the women, but not for the men. When the Bangladeshi asylum seekers were excluded, the association was no longer significant (*p* = 0.07); this may, however, indicate lack of statistical power. There was no significant difference in care certificates for compulsory care, but significantly more of the asylum-seeking women who were not referred to another hospital after the initial assessment had their compulsory care confirmed in a final admission decision. Asylum-seeking women also received a greater variation of types of psychotropic medication (neuroleptics, antidepressants, benzodiazepines and other hypnotics).

Significantly fewer asylum seekers than controls were referred to outpatient mental health services for follow-up, and significantly more were referred to primary care. (For more information, see Table 7.)

**Table 7:** Care interventions and treatment during registered care episode (*n*, %, range, median)

Intervention	Men, asylum seekers	Men, control group	Women, asylum seekers	Women, control group	Total, asylum seekers	Total, control group
Hospitalization, referral for hospitalization	42 (89.4)	40 (85.1)	37 (90.2)*	26 (63.4)	79 (89.8)*	66 (75.0)
Range of days (md) in hospital	1–167 (3.0)	1–136 (12.0)	2–197 (22.0)	1–157 (2.0)	1–197 (10)	1–157 (4.5)
Days in hospital, mean (standard deviation)	28.7 (46.1)	25.24 (35.1)	37.14 (43.8)	19.57 (40.2)	22.65 (37.2)	33.3 (44.6)
Psychiatric care certificate for compulsory care	25 (53.2)	27 (57.4)	26 (63.4)	18 (43.9)	51 (58.0)	45 (51.1)
Admission decision for compulsory care	8 (17.0)	8 (17.0)	17 (41.5)*	5 (12.2)	25 (28.4)^*^	13 (14.8)
Medication	25 (53.2)	29 (61.7)	28 (68.3)	25 (61.0)	53 (60.2)	54 (61.4)
Talking interventions/therapies	3 (6.4)	6 (12.8)	5 (12.2)	4 (9.8)	8 (9.1)	10 (11.4)
Contacts with other authorities	14 (29.8)	10 (21.3)	24 (58.5)*	11 (26.8)	38 (43.2)*	21 (23.9)
Referral to outpatient psychiatric care	7 (14.9)	21 (44.7)*	13 (31.7)	23 (56.1)*	20 (22.7)	44 (50.0)*
Referral to primary care	7 (14.9)*	1 (2.1)	12 (29.3)*	1 (2.5)	19 (21.6)*	2 (2.3)

* = significant difference, *p*-value <0.05

## Discussion

This study is among the first to examine a broad range of social risk and protective factors and factors related to previous and current mental health problems as well as characteristics of care in a population of asylum seekers. When comparing the asylum seekers and the control group we found several differences: different patterns of risk and protective factors, different clinical pictures and diagnoses and different treatment and follow-up.

One result that differed from most earlier studies on attempted suicide was that a majority of the attempters were men. This reflects the fact that a majority of the asylum seekers in Sweden during this period were men [38–40].

Our results suggest that the decision on the asylum application was an important risk factor, whereas some other studies have failed to find a relationship [2, 5]. The fact that almost a quarter of the studied sample of asylum seekers made a suicide attempt early in the process does not exclude stress related to the insecurity of the asylum process. Other studies have reported the asylum process as an important stressor irrespective of time of rejection [2, 7, 19, 21–26]. Combined with the fact that many of the asylum seekers had earlier mental health problems, the early suicide attempts could also indicate a more longstanding illness process and effects of premigration stress.

We found contrasting patterns of risk factors such as trauma and stressful situations in the two groups. The fact that 23 % of the asylum seekers had been exposed to torture is in line with demographic data from the UK [4] and with other studies on asylum-seeking suicide attempters [3, 6]. One circumstantial indicator of suicidal intent, carrying out the suicide attempt without anyone else present, was lower for the asylum seekers. On the other hand, two subjective indicators of intent - expressing the seriousness of the attempt and that its purpose was death - were higher for the asylum seekers. Discrepancies between these two sections are known in literature. The subjective section consistently generates higher intent scores. Freedenthal [31] discusses the possibility that suicide attempters exaggerate their intent in order to justify their actions, win attention or otherwise give socially desirable responses. In our study it may be thought that, in the case of asylum seekers being assessed within a special legal framework, there are incentives to exaggerate the suicidal intent. Freedenthal’s conclusion is that there may be other explanations, for instance that the objective section is less internally consistent, and that more qualitative research is needed to clarify these discrepancies.

There were significant differences in the diagnoses of stress reactions, PTSD, adjustment and personality disorders as well as alcohol and substance abuse. The levels of diagnoses of PTSD and stress reactions vary in studies of asylum seekers. Our group placed themselves in between the extremes of other studies [3, 4]. The observed low level of alcohol and substance abuse coincides with findings in a UK study [4].

Our data could not clarify how traumatic experiences were taken into account and influenced diagnostic procedures. Other studies have shown that an assessment using a cultural formulation has led to changes in diagnoses, particularly regarding PTSD and psychosis [43, 44]. Our aim is to deepen our understanding of the process of assessing trauma through a qualitative study of the same material.

Our study showed differences between the male and female asylum seekers that have not been demonstrated previously: The asylum - seeking men appeared more anonymous than the women and significantly more so than the male controls, since there was less documentation on their social context, on previous suicidal behaviour and on suicide in the family and close environment. This lack of exploration may affect the assessment of suicide risk. Further, exploring the individual’s social network is a way to approach the subjective world of the patient, which is essential in learning about the determinants of suicide. In this context, it is noteworthy that information on suicidal intention was lacking in a majority of the subjects in both groups.

The female asylum seekers more often made the suicide attempt closer in time to the asylum decision and they were more traumatized than the asylum-seeking men. Compared to the female controls they significantly more often used a suicide method classified as self-injury, regarded as reflecting higher suicide intent than self-poisoning [42]. They also received different diagnoses and more intense and prolonged treatment in hospital.

In spite of this, and in spite of the similarities in the previous history of mental health problems and suicide attempts, the asylum seekers as a group were referred to less specialized mental health care for follow-up than the controls. Staff within the mental health services might not find this surprising, since asylum seekers only had access to “treatment that cannot wait”. In official guidelines, however, suicidal ideation and behaviour are considered serious conditions that require attention by a specialist. Further, as early as 1995 official guidelines emphasized that intense anxiety or depression and torture-related problems should be thoroughly considered when the clinician makes a decision about whether care can wait or not [45].

The fact that the documented clinical picture, the diagnoses and the treatment interventions varied could have different interpretations: there could be a real difference in clinical problems; there could be differences in the assessment of the patient based on the clinician’s assumptions and understanding of cultural factors; more days in hospital could indicate a more serious condition or a lack of outpatient resources for this group of patients. Was trauma more often recorded in the asylum seekers simply because they more often disclosed trauma, given the importance that getting recognition for trauma may have in the asylum process? Or was it because the clinician asked them more often about trauma?

An additional finding is that the Bangladeshi asylum seekers had a higher representation in our study than among asylum seekers in general in Sweden in these years [46]. However, as there are no regional statistics of asylum seekers, we cannot be certain that they were overrepresented in our (Stockholm County) sample during the study period. If there is an overrepresentation, it is improbable that this reflects the suicide pattern of the home country [47]. Other possible explanations could be that Bangladeshi asylum seekers had a higher rate of rejection of their asylum claims and longer waiting time than the average applicant during the study period [48], that the Bangladeshi asylum seekers had different background trauma, and possibly greater difficulties than others in making themselves understood in the Swedish health care system.

### Strengths and limitations

The strength of the study is that we were able to access all registered suicide attempts during the research period. During this period, suicide risk identification routines that involved the systematic and mandatory registration of all suicide attempts were implemented. Given the paucity of data on asylum seekers our material is unique.

A limitation is that in spite of the guidelines we cannot know to what degree attempted suicide in the population was in fact registered.

Another strength is that using the medical records as a source made it possible for us to map a broad range of risk factors as they were perceived by the clinician and link them to the actions of the clinician. The obvious corresponding limitation is that we could not find information in medical records on some of the initially formulated items, reducing the number of variables that could be analysed. It was especially difficult interpreting absence of information, since medical records are not written systematically denying absent phenomena. Did the absence of a criterion indicate it not being present in the medical history, not being explored or not being documented?

Also we did not register a number of known risk factors for suicide in our protocol, including socioeconomic status and sexual orientation, since it was unlikely that such data would be documented in the medical records.

Another limitation is that the sample was too small to reach statistical power in some subgroup analyses, for instance the analysis of differences in suicide methods.

## Conclusions

The study shows clear differences in patterns of risk and protective factors and in clinical pictures. This illustrates the need for the assessing clinician to have a broad understanding of variations of risk factors and to avoid the stereotyped view of the expected suicidal person.

The study highlights a lack of early and thorough exploration of suicide intent for both groups and contextual and subjective factors for asylum seekers.

Training on these issues is needed for clinicians who meet and assess mental health and suicidality in general as well as in asylum seekers, in primary care as well as in psychiatry.

Regarding treatment, female asylum seekers received significantly different treatment than the female controls. The finding that the asylum seekers were referred to less specialized follow-up cannot be explained by differences in clinical picture or in suicide risk assessment and consequently indicates unequal access to care. More research is needed to understand the special needs of female asylum seekers and health care providers need to consider their local guidelines in order to ensure equal access to care.

The importance of the study is that it adds essential information on a group of help-seeking persons about whom there is a lack of systematic research. The results can help inform clinicians on important aspects in the encounter with the asylum - seeking patient.

## Abbreviations

DSM-IV: Diagnostic and Statistical Manual of Mental Disorders. Fourth edition
ICD-10: International Classification of Diseases. Tenth edition
PTSD: Post-Traumatic Stress Disorder
SIS: Suicide Intent Scale
